# Hyperspectral Face Recognition with Adaptive and Parallel SVMs in Partially Hidden Face Scenarios

**DOI:** 10.3390/s22197641

**Published:** 2022-10-09

**Authors:** Julián Caba, Jesús Barba, Fernando Rincón, José Antonio de la Torre, Soledad Escolar, Juan Carlos López

**Affiliations:** Technology and Information Systems Department, School of Computer Science, University of Castilla-La Mancha, 13071 Ciudad Real, Spain

**Keywords:** facial recognition, hyperspectral compression, hyperspectral imaging, biometrics, SVM, computer vision

## Abstract

Hyperspectral imaging opens up new opportunities for masked face recognition via discrimination of the spectral information obtained by hyperspectral sensors. In this work, we present a novel algorithm to extract facial spectral-features from different regions of interests by performing computer vision techniques over the hyperspectral images, particularly Histogram of Oriented Gradients. We have applied this algorithm over the UWA-HSFD dataset to extract the facial spectral-features and then a set of parallel Support Vector Machines with custom kernels, based on the cosine similarity and Euclidean distance, have been trained on fly to classify unknown subjects/faces according to the distance of the visible facial spectral-features, i.e., the regions that are not concealed by a face mask or scarf. The results draw up an optimal trade-off between recognition accuracy and compression ratio in accordance with the facial regions that are not occluded.

## 1. Introduction

Face recognition is a special branch of biometrics concerned with face identification. This is considered an easy task for humans but a challenge for the machines tasked with automatic face recognition. Traditionally, this process has been performed through an analysis of face features in which computer algorithms pick out specific, distinctive details about a person’s face. These details, such as distance between the eyes or mouth, are then converted into a mathematical representation (face encoding vector) and compared to other faces previously collected in a database [1]. In recent times, computer vision applications have been highly engaged via deep learning techniques. On this basis, most recent works advocate for the use of neural networks for face recognition, whose results are very promising [2].

Although face recognition is no longer considered a challenge due to the good results obtained by a variety of techniques and algorithms published in the scientific community, this topic has returned to the limelight where the scenario is not the usual one, e.g., in scenes where some details of the face are hidden. In this sense, the outbreak of the COVID-19 pandemic has introduced a new way of life for many, e.g., the use of face masks in public and private places, such as public transport, is mandatory in some countries or workplaces according to restrictions imposed by health authorities, mostly based on the status of virus transmission. However, the use of face masks compromises security due to their ability to conceal identifying facial features, e.g., criminals can exploit the new widespread use of face masks to veil their identity.

This fact opens up a new challenge in face recognition research, where traditional state-of-the-art approaches lack essential information, hidden behind masks, that would allow them to achieve successful results. It is worth mentioning that this challenge did not arise solely from the mask-related policies of the COVID-19 pandemic, since people generally wore clothing accessories prior to the pandemic, such as scarves or sunglasses, resuting in the same effect; the face is partially hidden. Thus, new facial features must be extracted from faces to fill this lack of information, e.g., encoding micro-expressions extracted from the facial regions of interest, as presented by Y.J. Liu et al., in [3], or extending the RGB information provided by conventional cameras through the use of wider spatial information obtained from hyperspectral sensors [4].

Hyperspectral imaging initially found its applications for remote sensing owing to the richness of the spectral information. This allows for the application of techniques with greater visibility in the thorough analysis of land surfaces through the identification of visually similar materials and the estimation of physical parameters of many complex surfaces [4]. However, apart from the spectral information captured by hyperspectral sensors, it complements the data information collected by traditional sensors, such as RGB cameras. These kinds of sensors have improved over the last decade by reductions in their cost and increases in imaging speed, which in turn has opened up hyperspectral imaging to other applications, and making it more popular than ever in recent decades [5,6]. Hyperspectral imaging is widely used for a large variety of applications such as precision agriculture, forestry, city planning, urban surveillance and homeland security, chemistry, forensic examination and face recognition.

In recent years, masked face recognition has gained great importance due to COVID-19, which is reflected in the number of articles published on this topic [7]. In this sense, there are many works on face mask detectors that trigger an alarm when detecting a person not wearing a mask or to analyse the degree to which health restrictions are enforced. On this basis, deep learning models have been used to automate the process of face mask detection. G.J. Chowdary et al. [8] have employed transfer learning of InceptionNet through augmented techniques to increase the diversity of the training data, as well as increase the performance of the proposed model. M. Loey et al. [9] have developed a hybrid deep transfer learning model that consists of two components for face mask detection; a component for feature extraction using ResNet50 [10] and a second component to classify face mask using decision trees and ensemble algorithm.

YOLO-based algorithms have also been used for face mask detection purposes, in which YOLOv3 is considered a major breakthrough in terms of the trade-off between detection precision and speed. S. Singh et al. [11] propose an efficient real-time deep-learning-based technique to detect masked faces by using YOLOv3 architecture that has been trained by a small custom dataset, in which authors have provided the necessary labels and annotations. T. Q. Vinh and N. T. N. Anh [12] present an algorithm comprising a Haar cascade classifier that detects the faces in a picture and whose output feeds the YOLOv3 algorithm that determines whether a person is wearing a mask. To do so, the YOLOv3 has been previously trained with the MAFA dataset. Other works go one small step further and detect more than just whether a person is wearing a face mask. P. Wu et al. [13] propose a YOLO-based framework to monitor whether people wear masks in a right mode, where the feature extractor, feature fusion operation and post-processing techniques are all specifically designed. Meanwhile, X. Su et al. [14] propose an efficient YOLOv3 algorithm, using EfficientNet as the backbone feature extraction network and reducing the number of network parameters, for mask detection and classify them into qualified masks (N95 and disposable medical masks) and unqualified masks (cotton, sponge, scarves, …).

The research efforts in masked face recognition have increased since the COVID-19 pandemic by extending previous works related to face recognition or occluded face recognition methods. One of the approaches adopted to face this challenge consists of restoring the part hidden by the mask and then using a face recognition alternative. In this sense, N. U. Din et al. [15] break the problem into two stages; firstly, a binary segmentation of the mask region is performed and then the mask region is replaced with face textures retaining global coherency of the face structure. To do so, authors use a GAN-based network with a discriminator that learns the global structure of the face and another discriminator that comes in to focus learning on the deep missing region. Unfortunately, these kinds of solutions result in failure when the map module is unable to produce a reasonable segmentation map of the mask object, i.e., the mask objects are very different than those in the dataset. These kinds of approaches follow the same strategy as older works in which a restoration process from a gallery takes place [16,17].

Other approaches only employ the visible part of the masked faces, i.e., these works extract the facial features from the upper part of the face or apply a filter to remove the mask area. W. Hariri [18] extracts deep features from the unmasked face regions through the last convolutional layer of three pre-trained deep CNN (VGG-16, AlexNet and ResNet50). A bag-of-features paradigm is subsequently applied to quantize the obtained features and, thus, a slight representation is obtained to, finally, feed a Multilayer Perceptron, that performs the classification process. F. Boutros et al. [19] propose an Embedding Unmasking Model (EUM) operated on top of existing face recognition models, such as ResNet50 [10] or MobileFaceNet [20]. These models do not require any modification or extra training. To do so, authors propose a loss function to guide the EUM during the training phase, minimizing and maximizing the distance between genuine and impostor pairs, respectively.

The lack of masked faces in well-known datasets has been managed by extending them with fake versions that contain masks, i.e., synthetic masked faces are generated from existing faces. Moreover, some proposals also enrich the datasets through data augmentation to create variations in the images, such as cropping, flipping or rotation. Thus, A. Anwar and A. Raychowdhury [21] combine the VGG2 dataset [22] with augmented masked faces and train the model following the original pipeline described in FaceNet [23]. This approach is also able to determine a masked face on the basis of the extracted features.

Despite the fact that hyperspectral imaging has not played a major role in face recognition owing to the success of other techniques, there are several works with a variety of techniques that address this problem. M. Uzair et al. [24] use an algorithm based on spatiospectral covariance for band fusion to merge hyperspectral images into one, and propose the Partial Least Squares (PLS) regression algorithm to achieve face recognition and classification. In addition, authors perform band selection experiments to find the most discriminative bands in the visible and near-infrared response spectrum. This band selection is followed by S. Bhattacharya et al. [25], where they propose a face-specific band selection framework to identify the optimal band set that results in satisfactory face recognition performance. Similarly, Q. Chen et al. [26] place emphasis on designing an efficient band selection method to reduce the spectral information without loss in recognition accuracy. V. Sharma et al. [27] propose hyperspectral CNN for image classification and band selection, where each band of the hyperspectral image is treated as a separate image. The architecture of the CNN is composed of six layers: three convolutional layers followed by two fully connected layers which are then connected to C-way softmax layer.

Pan et al. [28] studied the reflectance of skin tissues for face recognition by analyzing near-infrared spectral bands (0.7–1.0 μm), which vary individually, thus these bands are employed for human recognition. In this sense, the problem of luminance affecting face recognition is decreased by manually selecting five facial regions of interest: hair, forehead, right and left cheeks and lips. However, the strong aspect of this work is that it can be used to recognize faces in the presence of changes in facial pose and expression. They also fuse the spatial information of the hyperspectral image, where each pixel in the fusion image is selected from a specific band in the same position, thus this method transforms a 3D hyperspectral image cube into a 2D image. In contrast, W. Di et al. [29] apply three techniques to analyze the efficiency over a different set of bands, from the whole bands to a single band, or, using a subset of bands. Thus, the three techniques comprise whole band ${\left(2D\right)}^{2}$ PCA, single band ${\left(2D\right)}^{2}$ PCA with decision-level fusion, and band subset-fusion-based ${\left(2D\right)}^{2}$ PCA, in which the latter two methods follow a simple efficient decision-level fusion strategy. Authors conclude that the set of bands from 0.53 μm to 0.59 μm provide the most significant feature information since such bands correspond to the activity of human skin and absorption and reflection characteristics of carotene, hemoglobin and melanin.

In this work, we present a novel algorithm for facial features extraction (HyperFEA) from hyperspectral images, using a combination of computer vision techniques through histogram of oriented gradients (HOG) and hyperspectral transformations, to face recognition. In addition, a set of adaptive and parallel Support Vector Machines (AP-SVM) has been designed to classify unknown individuals. Thus, the extracted spatial information supply the missing information that face masks or clothing accessories occlude. To the best of authors’ knowledge, this is the first time computer vision techniques have been applied to hyperspectral images for this purpose. The main contributions of this work are listed as follows.

- An algorithm that uses computer vision techniques to extract facial regions of interests for face recognition of hyperspectral images.
- A significant compression of the spatial information obtained from the facial regions of interest that maintains the uniqueness of the face hyperspectral signature.
- An adaptive and parallel Support Vector Machine tree to distinguish unknown individuals using only the visible regions of interests.
- An evaluation of the proposed model to analyze the recognition accuracy and an analysis of the similarity results.

The rest of the paper is organized as follows. In Section 2, the characteristics of the HyperFEA algorithm used in this work are introduced in detail to understand the extraction process of face features from hypersperctral images and the set of SVMs is also depicted. Section 3 describes the hyperspectral data sets, the performance assessment metrics used to evaluate the accuracy of the results provided by the proposed algorithm and shows the experimental results. Section 4 compares the results with those obtained for other state-of-the-art proposals. Finally, Section 5 draws up the main conclusions of this work.

## 2. Materials and Methods

### 2.1. Extracting Spectral Information

Feature extraction is a crucial stage in face recognition, whose main objective is to obtain a set of features that clearly represent a person. Typically, the set of features is composed by key facial attributes, such as eyes, mouth or nose, and/or the distance between them. From these features, a face encoding vector is generated and used to determine a similarity measure with other individuals in the recognition process. Unfortunately, the feature extraction process becomes more complicated when people wear face masks or clothing accessories, i.e., part of the key facial features become hidden. This fact leads to the adaptation of existing face recognition methods in order to extract representative facial features. On this basis, we propose the use of spectral information to replace the information lost. Thus, the proposed algorithm, HyperFEA extracts the relevant spatial information from hyperspectral faces. This algorithm has been developed for providing a good recognition accuracy by extracting facial regions of interests as well as providing a good compression performance of the spatial information of such regions. Additionally, the algorithm follows an unmixing-like strategy which selects the image pixels that are potentially more useful.

**Figure 1:**
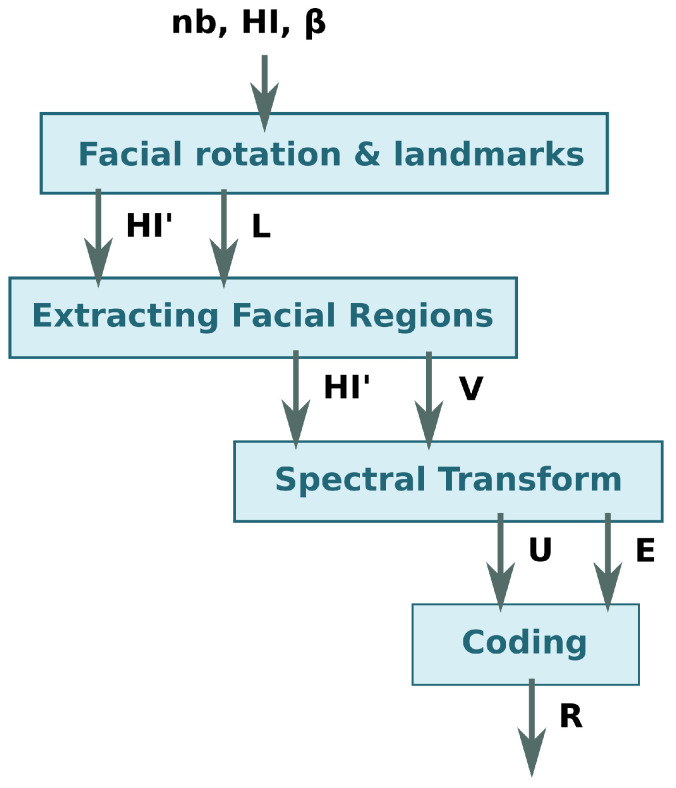
Diagram of the HyperFEA algorithm stages.

The process performed by the HyperFEA algorithm to hyperspectral images consists of four main stages, which are: (1) facial landmarks stage, which extracts the points where a face is located and the face is optionally rotated horizontally to align it; (2) extracting facial regions of interests (ROI) where the unused spatial information is removed; (3) a spectral transform; (4) a coding stage. The HyperFEA spectral transform selects the most different pixel and the average pixel of each facial ROI. Figure 1 graphically shows these four stages, as well as the data shared between them.

#### 2.1.1. Algorithm Notations

In the following, $\mathrm{HF}=\left\{{\mathrm{HI}}_{i},i=1,...,ns\right\}$ is a sequence of ns hyperspectral frames, ${\mathrm{HI}}_{i}$, comprised by nb spectral bands that represent a hyperspectral image. Whereas ${\mathrm{HI}}^{\prime }$ is the aligned hyperspectral image obtained from HI whose maximum deviation of the angle formed by the eyes is set by β (depicted in degrees). $L=[l_{1},l_{2},...,l_{\alpha }]$ represents the facial landmark points, where α is the number of landmarks. Whilst $V=[V_{1},V_{2},...,V_{p}]$ depicts the position of the points that delimits the facial regions of interests extracted from L, where *p* is the number of regions; i.e., $V_{i}$ corresponds to the points that set the limits of the *i* facial ROI. ${\mathrm{HR}}_{i}$ represents the *i* hyperspectral region whose location is stored in $V_{i}$. The average pixel, also called centroid, is represented by the symbol $\hat{\mu }$, while $\hat{e}$ represents the most different hyperspectral pixel extracted from such region. Each facial ROI can be represented as $R_{i}=\left(\hat{e},\hat{\mu }\right)$.

Therefore, in addition to the hyperspectral image containing the face, the HyperFEA algorithm employs two main input parameters to extract the facial ROI and the spectral information.

- Number of bands (*nb*). This parameter denotes the number of bands that contains the hyperspectral images. It is provided to the algorithm in order to consider the whole spectral information.
- Degrees threshold (β). It determines a threshold of degrees up to the hyperspectral image must be rotated, i.e., all bands are rotated until they all fulfil this requirement.

#### 2.1.2. HyperFEA Algorithm

The HyperFEA algorithm is described in detail in Algorithm 1 for a hyperspectral image, HI. Firstly, the hyperspectral face is rotated (${\mathrm{HI}}^{\prime }$) and, then, the facial landmarks are extracted from it, L, in lines 1 and 2, respectively. From the location of the face landmarks, the algorithm infers the facial ROI by obtaining the matrix V that contains the area of each region ($V_{i}$), i.e., $V_{i}$ represents the cloud of points that delimits the *i*th facial ROI. The hyperspectral facial ROI is obtained from ${\mathrm{HI}}^{\prime }$ by cropping the image according to the set of points stored in $V_{i}$ (line 5 of Algorithm 1). Thus, the algorithm calculates the centroid and the brightest pixel of each hyperspectral facial ROI, ${\mathrm{HF}}_{i}$. The average pixel or centroid (${\hat{\mu }}_{i}$) is computed in line 6. Afterwards, the facial ROI is centralized by subtracting the average pixel to the original spectral information, i.e., each hyperspectral pixel that contains the facial ROI is subtracted by the average pixel (see line 7 of Algorithm 1). In addition, the most different pixel is extracted in line 11. In the remainder of this document, it is referred as brightness of a pixel. In this process, the dot product of each frame pixel within the centralized facial ROI with itself is first computed (lines 8 till 10 of Algorithm 1), whose the maximum value corresponds to the highest brightness (${\hat{e}}_{i}$). Finally, both spatial features, ${\hat{\mu }}_{i}$ and ${\hat{e}}_{i}$, are stored in the matrix R in line 12, which contains the whole spatial information extracted from a hyperspectral face and it will be used to compare it with other matrix and determine its similarity.
**Algorithm 1** HyperFEA algorithm.   **Inputs:**   $\mathrm{HI}=[b_{1},b_{2},...,b_{nb}]$, nb, β   **Outputs:**   $R=\left[R_{1},R_{2},...,R_{\alpha }\right];R_{i}=\left({\hat{\mu }}_{i},{\hat{e}}_{i}\right)$
   **Algorithm:**
1:Face Alignment: ${\mathrm{HI}}^{\prime }=\left[b_{1},b_{2},...,b_{nb}\right]$;2:Facial Landmarks: $L=[l_{1},l_{2},...,l_{\alpha }]$;3:Location of Facial ROI: $V=[V_{1},V_{2},...,V_{p}]$;4:**for** *i* **in** *V* **do**5:   Get hyperspectral region: ${\mathrm{HR}}_{i}\leftarrow$  $getRegion({\mathrm{HI}}^{\prime },V_{i})$;6:   Centroid or average pixel: ${\hat{\mu }}_{i}$;7:   Centralization: $C={\mathrm{HR}}_{i}-{\hat{\mu }}_{i}$;8:   **for** *j* **in** $HR_{i}$ **do**9:      Brightness Calculation: $b_{j}=c_{j}^{\prime }\cdot c_{j}$;10: **end for**11: Maximum Brightness: ${\hat{e}}_{i}=\mathrm{argmax}\left(b_{j}\right)$;12: Save Spatial information: $R_{i}\leftarrow$  (${\hat{\mu }}_{i}$,${\hat{e}}_{i}$);13:**end for**

#### 2.1.3. Face Alignment and Extracting Facial Landmarks

**Figure 2:**
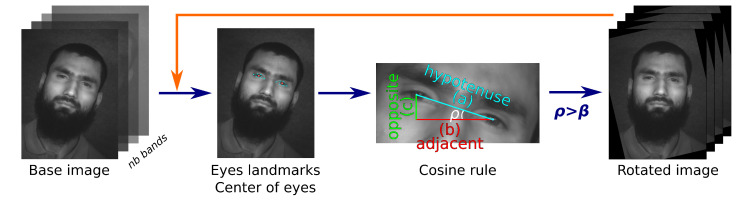
Face alignment process.

Face alignment is an early stage of the modern face recognition pipeline that increases the recognition accuracy, which is optional in our proposal. Figure 2 shows the steps performed in the face alignment process. The first step is to detect the location of the eyes to extract the center of them and imaginatively draw a line between the two centres. Thus, the angle formed by the horizontal line with the previously one (ρ) gives the degree of inclination of the face. From this angle we can determine the rotation degrees by applying inverse trigonometry functions (*arc cosine* function), the result in degrees determines the angle to rotate the image, whenever its value is greater than β, i.e., the threshold degree parameter. Once the image is rotated, the algorithm checks that the face is horizontally aligned by a new iteration, it means the rotated image is the new input (orange arrow of Figure 2). In general, the constraint is fulfilled in the first iteration, which is an important issue when working with hyperspectral images, due to the computational cost required; the operations of the rotation stage are applied to all bands, i.e., the face alignment stage is repeated at least *nb* times. Thus, the hyperspectral face is horizontally aligned, ${\mathrm{HI}}^{\prime }$ (line 1 of Algorithm 1).

From a trigonometry point of view, the algorithm draws a rectangular triangle to calculate the angle between eyes (see multi-color triangle of Figure 2), whose sides correspond as follows: line between the centers of the detected eyes (hypotenuse, blue line), horizontal line between the center of the detected eyes (adjacent, red line) and the line that closes the triangle (opposite, green line). The length of the three lines are calculated with Euclidean distance algorithm from the 2D points of the three edges of the triangle. Then, the cosine rule (see Equation (1)) is performed to obtain the ρ angle.

(1) $$cos\left(\rho \right)=\left(b^{2}+c^{2}-a^{2}\right)/2bc$$

The face alignment process can be carried out in two different modes depending on the speedup of the hyperspectral sensor to capture the images. The first mode considers each spectral band independent of the others, even if the captured face moves during exposure of the picture being taken, the alignment process corrects such small deviations. Meanwhile, the second mode takes as reference the first spectral band, which is aligned, and from the degree to which it is aligned, the rest of the bands are rotated. This mode is only suitable for those hyperspectral sensors whose time of exposure is small, i.e., can be omitted. Thus, the facial alignment process is independent of the hyperspectral sensor speedup feature, but is mandatory for hyperspectral sensors with long exposure times.

**Figure 3:**
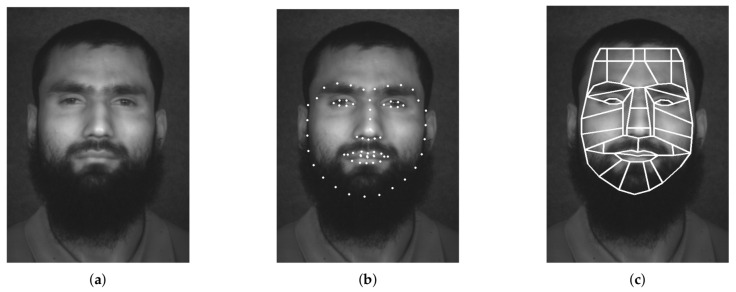
Extracting facial regions of interest from facial landmarks. (**a**) Base image. (**b**) Facial Landmarks. (**c**) Facial regions of interest.

After the horizontal facial alignment phase, the next step is to extract the facial landmarks by providing a set of cloud that contains 2D points. These points represent and localize salient regions of the face, such as eyes, eyebrows or nose (see Figure 3b). The process is divided in two steps; firstly, the face must be localized in the image and then the key facial structures are detected. This method is widely used in RGB or grayscale images, so it is suitable for hyperspectral images, where both operations, face localization and facial landmark detection, are performed using the first spectral band. The algorithm considers that the rest of bands are aligned, either because the facial alignment process has been applied or because the hyperspectral sensor is able to capture all the spatial information in a shot. The result of this stage is a dictionary of lists, L, where the location of α salient regions are stored (line 2 of Algorithm 1).

#### 2.1.4. Extracting Facial Regions of Interests

The next stage is to obtain the location of facial ROI (line 3 of Algorithm 1). This process is similar to that applied by F. Becattini et al. in [30], where 36 facial ROI are estimated. The solution proposed extracts 38 facial ROI from the location of the facial landmarks (L), which were obtained in the previous stage (see Figure 3c). The facial regions are represented by a set of 2D points, which corresponds to the *x*-axis and *y*-axis position within the hyperspectral image (${\mathrm{HI}}^{\prime }$). The 2D points that delimit the facial ROI are stored in a list (V) that will be used for the spectral transform process.

**Figure 4:**
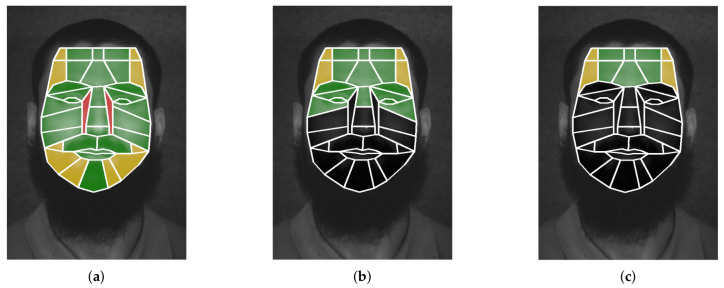
Quality of spectral information related to each facial ROI according to the visible parts. (**a**) Facial ROI no mask. (**b**) Facial ROI with mask/scarf. (**c**) Facial ROI with sunglasses and mask/scarf.

In turn, there are problems in capturing hyperspectral images caused by the position of the hyperspectral sensor, light exposure on the face and the shape of the face itself, i.e., the quality spatial information depends on the reflection of the light over the surface of an object, a face in our case, and also depends on the position of the hyperspectral sensor, meaning that the regions located in front of the sensor will have good quality spatial information. Thus, the shape of the face is not flat, it appears balloon-like in which the luminosity does not produce good reflection over all parts. This fact is the reason that some facial ROI are divided in order to obtain useful spatial information instead of including them as a single piece, e.g., the forehead is broken into ten subregions, where the lateral subregions do not provide good spatial information. Figure 4 shows the biometric facial areas of interest that have been extracted in accordance with the visible face parts. In addition, the spatial information quality of the collected facial ROI is also highlighted and classified as good (green), mid (yellow) and poor (red).

It is worth mentioning that clothing accessories and/or facial masks hide part of the face, so this work only takes the upper facial ROI for the experiments; from ten to nineteen regions. Figure 4b,c highlight the facial ROI considered, using the same colors that were used to mark the quality of the spatial information, whilst regions that are omitted are darkened.

#### 2.1.5. Spectral Transform

The spectral transform has a twofold objective: it contains enough information to discriminate between people and the spatial information is reduced. The second feature directly depends of the number of regions of interests and the number of spatial information, i.e., the number of bands.

Therefore, the HyperFEA transform sequentially selects the most different pixels ($\hat{e}$) and the average pixel or centroid ($\hat{\mu }$). For this purpose, a rectangular area of the hyperspectral image is extracted to apply a mask, which was previously generated from the set of 2D points of the facial ROI that is being computed ($V_{i}$). Therefore, the result is a hyperspectral image where the pixels out of the ROI have a value of 0, so spectral operations do not consider such pixels. Then, from each facial ROI a centroid is extracted (${\hat{\mu }}_{i}$) (line 6 of Algorithm 1) by computing Equation (2), where $p_{x,y}^{k}$ represents the pixel located in the kth band (spatial axis) at *x*,*y* position (*x*-axis and *y*-axis, respectively). The result is a hyperspectral pixel composed by *nb* bands whose values are the average value of each band.

(2) $${\hat{\mu }}_{k}=\frac{\sum_{x=0,y=0}^{x=W,y=H}p_{x,y}^{k}}{NPixels_{valid}}$$

Afterwards, the hyperspectral facial ROI is centralized by subtracting the centroid, i.e., the subtraction operation is applied between each pixel in the ROI and the centroid (line 7 of Algorithm 1). In turn, the most different pixel of such ROI (${\hat{e}}_{i}$) is obtained by calculating the brightness of each pixel (lines 8 to 10 of Algorithm 1). The brightness is obtained by applying the $l^{2}-norm$ vector normalization algorithm; it squares the root of the sum of the squared element of the hyperspectral pixel. Then, the highest brightness pixel is selected (line 11 of Algorithm 1).

**Figure 5:**
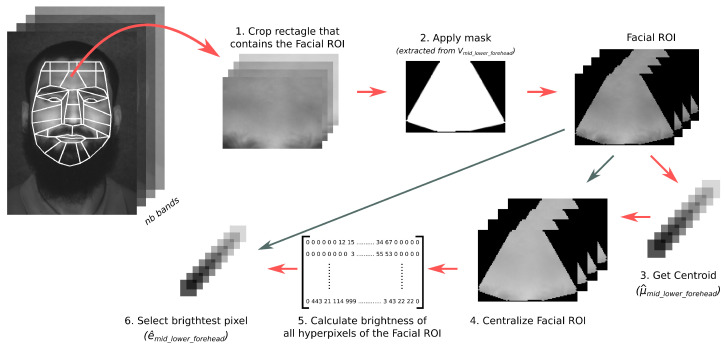
Flow of spatial information extraction process.

Figure 5 graphically shows the flow to extract the spatial information of the *mid_lower_forehead* facial ROI from a hyperspectral face. The algorithm extracts a rectangle that contains the hyperspectral pixels of the facial ROI to then apply a mask to crop the region, considering the pixels that are inside the region. Thus, the algorithm obtains the number of valid pixels and extracts the average value of each spectral band to build the centroid (${\hat{\mu }}_{mid\_lower\_forehead}$) and extracts the brightest hyperspectral pixel (${\hat{e}}_{mid\_lower\_forehead}$). It is worth mentioning that the spectral information extracted of each facial ROI can be performed in parallel.

### 2.2. A Tree Based on Adaptive and Parallel SVMs for Face Recognition

Cascade of Support Vector Machines (C-SVM) has been introduced as an extension to classic SVM devoted to accelerate inference time by using a horizontal scaling strategy. The concept relies on the division of the problem into smaller problems, where each layer of SVMs is considered as a filter. This way, it is straightforward to obtain partial solutions leading towards the global optimum [31]. On this basis, the proposed solution leverages the advantages of C-SVM by proposing an adaptive and parallel-layered solution (AP-SVM). AP-SVM includes layers that may contain two or more SVMs, which are trained at run-time with the output of the previous layer. The output of one layer denotes the elements of the dataset that the next layer of SVMs must be trained with. Since the size of the training dataset decreases as the pipeline advances, overall latency of the process does not soar.

**Figure 6:**
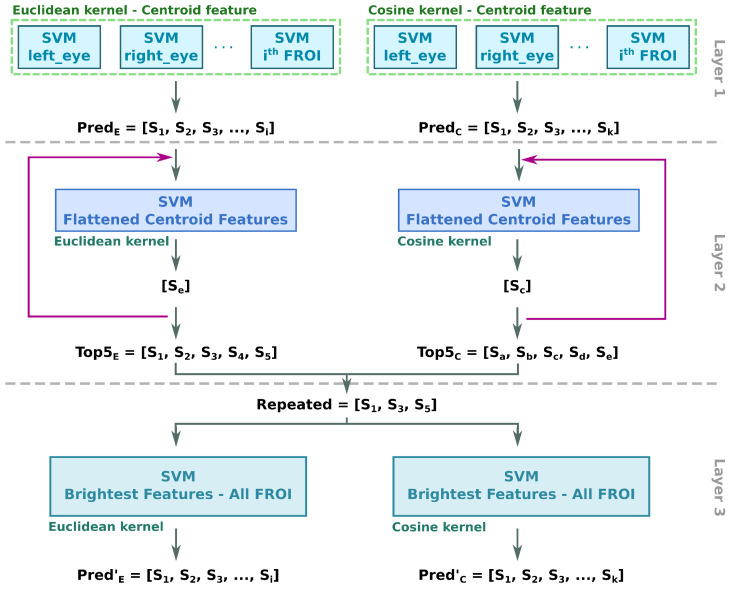
AP-SVM tree for face recognition.

Figure 6 shows the AP-SVM tree structure for face recognition purposes using hyperspectral images. Classification time is sped-up due to the parallelization, following the C-SVM approach. For example, in layer one there is one SVM per ROI and per SVM kernel used in this work. In addition to the independent and concurrent processing of each ROI, the size of the problem is smaller which leads to reduced latency.

Two kernels have been customized to obtain the closeness degree between the individual to be identified and the well-known persons in the dataset. AP-SVM uses the cosine similarity (i.e., computes the angle between two vectors) and Euclidean distance (i.e., calculates the distance between two points). The cosine similarity is used to model the affinity in the reflectance realm whilst Euclidean distance helps to model the spatial differences between concerning the morphology of the face.

#### 2.2.1. Layer 1: Centroid Classification

The main goal of the first layer is to obtain a set of subjects that are close to the unknown individual. To do this, the problem is divided into as many SVMs as the number of facial ROIs used (horizontal division). In addition, the split is duplicated, in accordance with the number of kernels used (Euclidean and cosine). The SVMs of this layer are previously trained and do not change during the classification process. The output of this layer is a list of potential candidates ($Pred_{E}$ and $Pred_{C}$ lists) composed by the subjects whom spatial signature for a particular region is the closest to the individual that is being classified.

#### 2.2.2. Layer 2: Flattened Centroid Classification

Once the first list of candidates is extracted by layer 1, it is necessary to measure the distance of the complete spatial signature between all the candidates and the unknown individual. For each ROI, the centroid and brightest pixels are selected and flattened in a single spatial features vector. Then, two SVM (one for each aforementioned kernel) are trained during run-time with the flattened spatial information vector in parallel. Afterwards, classification of the individual takes place. This process depends on the output of the previous layer, so the SVMs are trained every time an outsider is classified (adaptive feature). As a result, the two SVMs predict two possible candidate sets; the ones whose Euclidean distance and cosine similarity are the shortest, respectively, ($\left[S_{e}\right]$ and $\left[S_{c}\right]$). Whenever the size of the $\left[S_{e}\right]$ and $\left[S_{c}\right]$ lists are not equal, they must be extended. Finally, the second layer of the AP-SVM extracts the top five candidates of both SVMs ($Top5_{E}$ and $Top5_{C}$ lists).

This layer is also considered as the decision layer. The unknown individual can be considered identified anytime the candidate obtained by both SVMs is the same after the first iteration (i.e., $S_{1}=S_{a}$). This means that the candidate has the shortest cosine and Euclidean distance. The second rule for direct candidate selection establishes that if the first two candidates output by the Euclidean SVM are equal (i.e., $S_{1}=S_{2}$) that must be the identification of the unknown individual.

#### 2.2.3. Layer 3: Brightest Classification

If the unknown individual is still unable to be identified, the last layer proposes a unique candidate based on the analysis of the brightest features. Therefore, two SVMs are trained with the $Top5_{E}$ and $Top5_{C}$ candidate lists using the brightest features solely. In this case, the brightest features are considered in the same pool for the Euclidean and cosine SVMs because the brightest features can be repeated or located in different facial ROI.

## 3. Experimental Results

**Figure 7:**
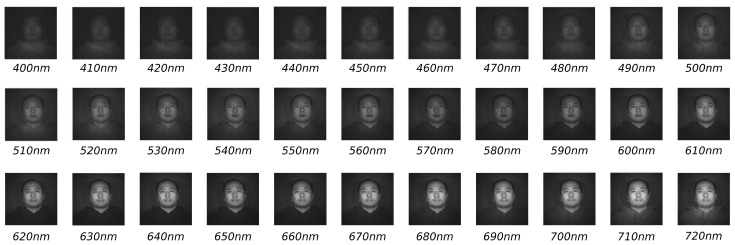
UWA-HSFD: Example of a subject’s face cube.

In this section, the hyperspectral data used for evaluating the recognition accuracy of the proposed model are introduced. The hyperspectral dataset used in experiments has been provided by The University of Western Australia through a database that consists of 164 facial hyperspectral images of 79 different subjects (UWA-HSFD), where 75 individuals are males and 4 females; of those 75 males, 13 wear glasses, while in the case of females, only one wears glasses. The face database was sensed by CRI’s Varispec LCTF, equipped with a photon focus camera helping in adjusting exposure time, luminance adaption and CCD sensitivity. Image cubes were captured over 33 bands from 0.4 μm to 0.72 μm with a difference of 0.1 μm; each band is stored in separate files. Figure 7 shows an example of a subject’s face cube with the 33 bands [24]. The hyperspectral images have been organized in four sessions, i.e., the repeated faces were taken on different days. Unfortunately, this dataset presents an important challenge; some subjects did not keep their head still during the process of capturing the image cube, so there are variations in the dataset.

To extract the spectral signatures of the images using the proposed method presented in this work, we have set the maximum error of the face horizontal alignment to one degree, which has been applied to all spectral bands. This configuration requires high computational costs; the extraction of a spectral signature required an average of 248 s on an i7-10710U CPU with 32 GB of RAM and SDD, i.e., the process to obtain all spectral signatures took 9 h and 47 min. It is worth mentioning that this stage can be optimized in performance terms; the horizontal alignment of each band can be carried out in parallel and each facial ROI could also be independently processed.

### 3.1. Partial Results of the AP-SVM Tree

**Figure 8:**
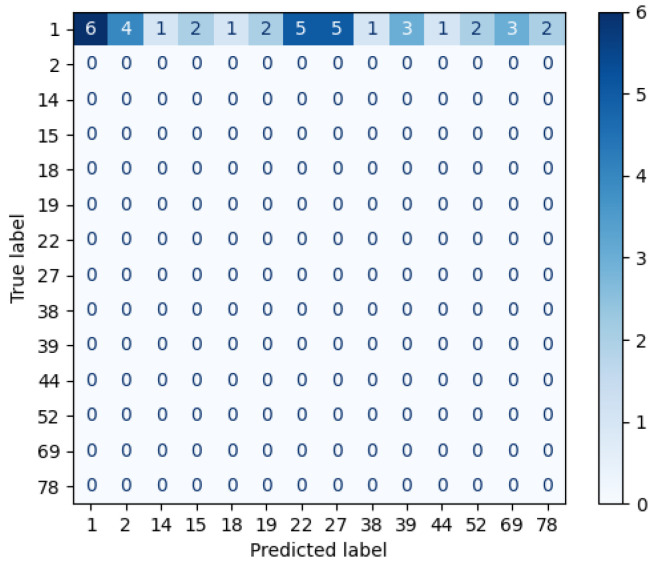
Example of confusion matrix obtained after the classification of layer 1.

For the sake of clarity, this section describes through a case study the intermediate results of the AP-SVM, using the 19 facial ROI highlighted in Figure 4b. The SVMs of the first layer are trained with the two first sessions, in which certain individuals appear in both sessions, thus the spectral information is doubled. The individual to recognize is labeled as 1 by using the spectral signature of the third session. Figure 8 displays the confusion matrix obtained after the classification performed by the SVMs of the first layer over the unknown individual. In this example, there are fourteen candidates; the subject 1 is the one where the distance of the spectral information is smallest. The SVMs of this stage are trained in parallel and, hence, it requires significant computational resources to optimize the time performance of the AP-SVM. Thus, this process takes up to 2 s.

**Figure 9:**
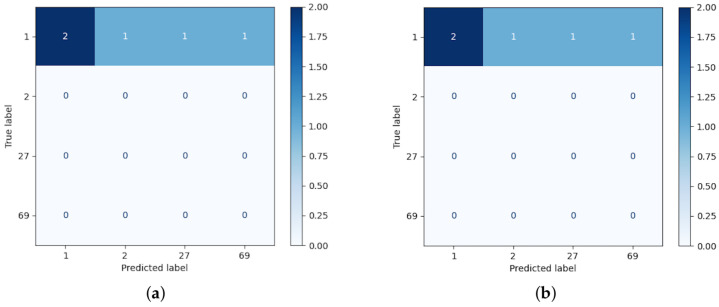
Example of separated confusion matrix obtained after the classification of layer 2. (**a**) Euclidean kernel. (**b**) Cosine kernel.

Although the subject 1 contains more spatial features, we cannot discard other candidates that contain similarities. Thus, the second layer measures the entire spectral information providing two *Top5* lists. In this case, the top of these lists are not equal in either the first or second item of the *Top5* related to the Euclidean distance. Figure 9a,b display the confusion matrices using the Euclidean and cosine custom kernels, respectively, which have been applied to the whole spectral signature. To obtain the list of candidates whose spectral signature is close to that of the unknown individual, the closest one is not considered in the next iteration. Thus, the list of candidates was obtained in the following order (2, 1, 1, 69, 27) and (1, 2, 78, 27, 27) for the Euclidean and cosine kernel, respectively.

The results draw differences between the two custom kernels, i.e., the candidates of the first step differs, the Euclidean kernel states that the unknown individual is the subject labeled as 2, whilst cosine kernel asserts that it is the subject 1, so the first rule to find out who is the unknown individual is not fulfilled. The second rule compares whether the first and second candidates of the Euclidean distance are the same, but this is not the case either.

**Figure 10:**
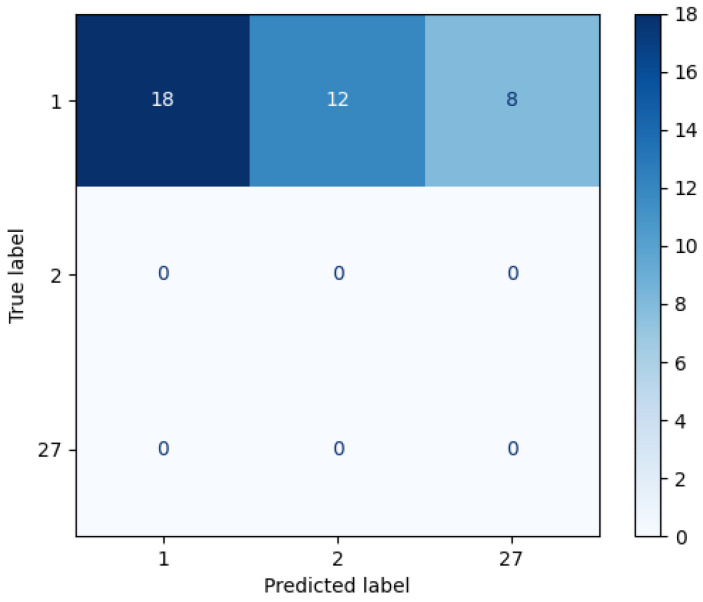
Example of confusion matrix obtained after the classification of layer 3.

Afterwards, the repeated candidates are the input of the third layer, which calculates the brightest cosine and Euclidean distance. Thus, the subjects (1, 2, 27) are considered to identify the individual. Figure 10 shows the confusion matrix after measuring the cosine and Euclidean distances of the brightest pixels. Therefore, the AP-SVM determines that the unknown individual is the subject 1, because it contains more similarities than the others, i.e., it has more coincidences in the brightest distance feature.

### 3.2. Facial Recognition Accuracy

The achieved facial recognition accuracy directly depends on the visible facial ROI and the classification performed by the first layer of the AP-SVM and the repeated items obtained from the second layer, i.e., the cosine and Euclidean *Top5*. Table 1 lists the *Top5* and *Top3* in percentage for the three following scenarios: when there are no objects that hide the face (100%), when an object, such as a scarf or a mask, occludes the lower part of the face (50%) and when the forehead is the only visible part (25%). Moreover, the aforementioned scenarios reduce the spectral information that can be extracted (see Figure 4) and, hence, the accuracy recognition achieved. In contrast, the compression ratio is greater and the computational costs are reduced. It is worth mentioning that the maximum recognition accuracy is as high as that obtained by the two first layers.

**Figure 11:**
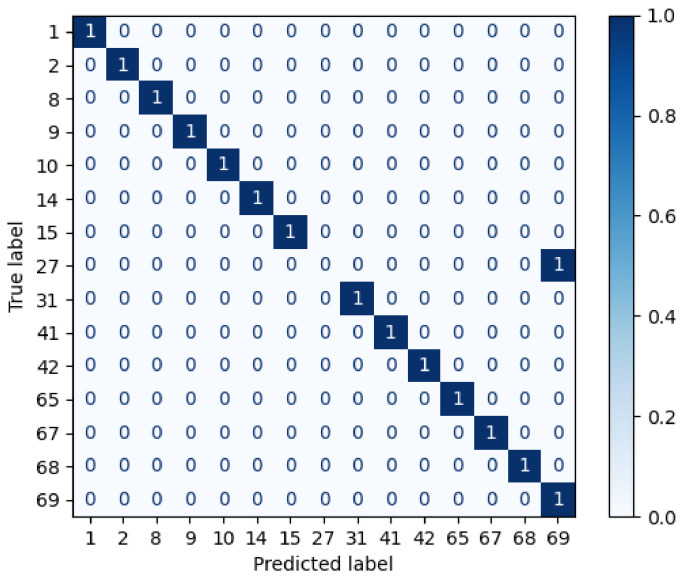
Confusion matrix with the 100% visible of the facial ROI.

The first scenario does not introduce objects that occlude any facial ROI, so there are 36 visible regions to extract the spatial information (see Figure 4a). In total, 72 features are obtained corresponding to the centroids and the brightest pixels of each of the visible areas. This fact reduces the spectral information by roughly 99.9933% that is used to classify the unknown individuals. Figure 11 shows the confusion matrix in this scenario, in which the recognition accuracy achieved is 93%, i.e., 14 out of 15 individuals are recognised by the AP-SVM.

**Figure 12:**
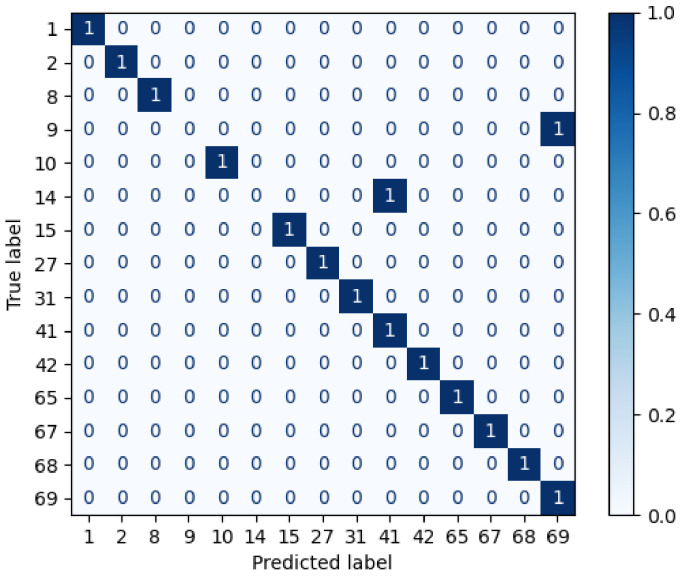
Confusion matrix with the 50% visible of the facial ROI.

The second scenario only makes visible the facial ROI located in the upper part of the face (see Figure 4b). Thus, 19 facial ROI are used to extract the hyperspectral signature composed by 38 features, reducing the information by roughly 99.9963%. The confusion matrix of the aforementioned scenario is shown in Figure 12; 13 of 15 individuals are recognized (86.67%). Due to the lack of a dataset that contains hyperspectral information with clothing accessories, such as scarves or sunglasses, we have only selected the facial ROI that are visible in the corresponding scenarios.

**Figure 13:**
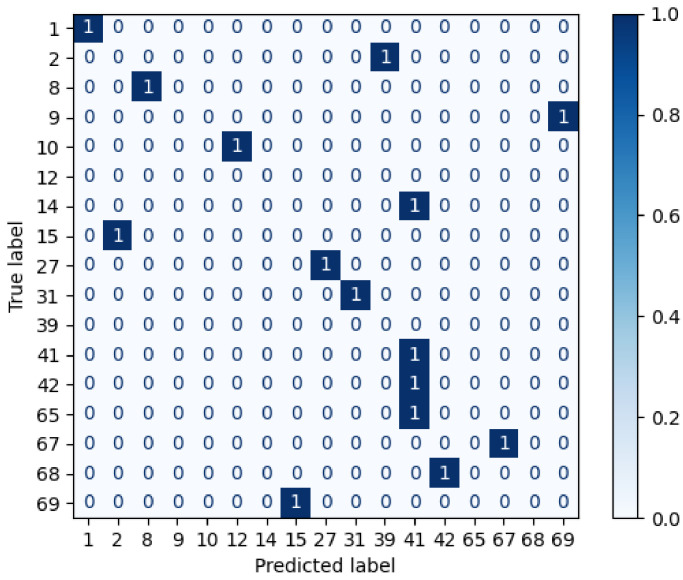
Confusion matrix with 25% visible facial ROI.

The last scenario imposes the most visibility restrictions in which 10 facial ROI are visible, which match with the regions of the forehead (see Figure 4c). This lack of spectral information results in lower facial recognition accuracy; only 6 out of 15 individuals are recognized (40%) as is shown in Figure 13. In contrast, the spectral information stored is reduced by up to 99.9981%.

### 3.3. Performance Evaluation Metrics for the AP-SVM Tree

The performance of classification results has been exhibited through four key metrics such as precision, recall, f1-score and accuracy, whose calculation formulas are expressed in Equations (3)–(6), where *TP, TN, FP* and *FN* denote the true positive, true negative, false positive and false negative, respectively.

(3) $$Precision=\frac{TP}{TP+FP}$$

(4) $$Recall=\frac{TP}{TP+FN}$$

(5) $$F1\text{-}Score=2*\frac{Precision*Recall}{Precision+Recall}$$

(6) $$Accuracy=\frac{TP+TN}{TP+TN+FP+FN}$$

**Figure 14:**
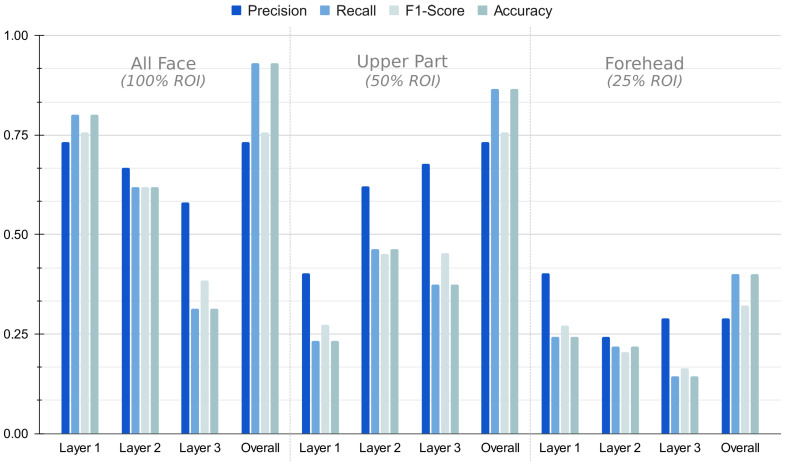
Performance metrics for the AP-SVM tree with different visible parts of the face.

These evaluation criteria on face classification have been applied in the three aforementioned scenarios: all face (100% ROI), upper face (50% ROI) and forehead (25% ROI). Figure 14 graphically shows the results of the evaluation metrics used in the different layers of the AP-SVM tree and on the overall AP-SVM. It is worth mentioning that *Recall* and *Accuracy* bear the same values, meaning the model is somehow balanced, i.e., the AP-SVM is able to correctly classify positive unknown individuals as well as correctly classify negative unknown individuals.

## 4. Discussion

The trade-off between compression ratio and recognition accuracy has been compared with five state-of-the-art proposals that use hyperspectral images. Table 2 shows the recognition accuracy obtained by different studies on hyperspectral face recognition as well as the compression ratio achieved by them. Z. Pan et al. [28] achieved a compression ratio of up to 99.9% because they manually selected five key regions for the frontal face corresponding to the forehead, left cheek, right cheek, hair and lips by achieving roughly 75% correct coincidences. Unfortunately, only two of these regions are visible when a person wears a mask or a scarf, so the accuracy recognition results will be worse. Similarly, W. Di et al. [29] manually locate the eyes’ position and from them the face is extracted, whose size is 162 × 150. Then, the extracted hyperspectral cube is normalized and scaled to 54×50 with the aim to save on computational costs. The rest of the works [24,27,32] also crop and resize the face area and perform an image fusion to transform the hyperspectral cube into a flattened image, which is obtained by band fusion. V. Sharma et al. [27] keep the whole spectrum range but the face is resized to 263×263 pixels, so the compression ratio is worse than that of other studies but the recognition accuracy is high. Meanwhile, authors of works [24,29,32] perform a band selection as well as resizing of the face area to reduce the spatial information obtaining high percentages of successful hits in facial recognition.

Therefore, the state-of-the-art proposals apply one of the two following methods with/without band selection: band fusion through calculating the average of each band or selection of a key pixel in a facial ROI. Nevertheless, HyperFEA algorithm automatically delimits the face area and its facial ROI without a band selection to extract the average pixel (centroid) and a key pixel (brightest pixel) of each region. On this basis, the face area is not resized to save computational costs.

**Figure 15:**
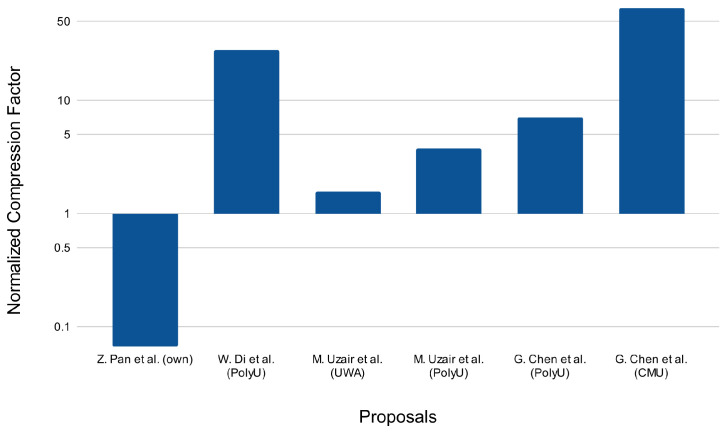
Normalized compression factor with respect to our proposal [24,28,29,32].

Figure 15 shows the compression factor normalized with respect to our proposal. For the sake of clarity, the proposal presented by V. Sharma et al. [27] has not be considered in this study due to the compression ratio being the poorest. The results draw up a good balance between recognition accuracy and compression ratio considering that our proposal is one that reduces the hyperspectral information the most.

The recognition rate obtained by our proposal has also been compared with other state-of-the-art proposals, whose objective is to recognize unknown individuals that wear masks or other clothing accessories that occlude part of the face. Table 3 lists the recognition rates achieved by some state-of-the-art proposals in the three aforementioned scenarios. Moreover, Table 3 also depicts the method used by each proposal. The results reveal that our proposal is close to the state of the art when the face is hidden behind a mask. Meanwhile, when the forehead is the only face region visible, the recognition rate of our solution doubles that proposed by A.C. Tsai et al. [33].

## 5. Conclusions

This work has focused on extracting features from hyperspectral images by using computer vision techniques and classification of unknown individuals through an AP-SVM tree. The process starts with the detection of the facial ROI. Then, the centroid or average pixel and the brightest pixel are extracted from each facial ROI. In contrast to works in the literature, which use hyperspectral images for face recognition, HyperFEA algorithm automates the extraction of spectral characteristics of a face. In addition, it facilitates the extraction of such features in parallel by using parallel-programming architectures, such as GPUs or FPGAs, where each facial subregion will be processed by a different kernel.

Experimental results reveal an interesting trade-off achieved by the HyperFEA algorithm, in which the compression ratio reaches up to 99.99% and the recognition accuracy is 93%, when all facial ROI are visible. However, the results are more interesting when several regions of the face are hidden by objects, such as masks, sunglasses or scarves, where the recognition accuracy achieved reaches up to 86.67%. This result could be improved by using hybrid hyperspectral and non-hyperspectral techniques in circumstances where they are self-complementary.

## Figures and Tables

**Table 1:** *Top5* and *Top3* results obtained from the second layer of the C-SVM (depicted in percentage).

Top	All Face (100% ROI Visible)	Upper Part (50% ROI Visible)	Forehead (25% ROI Visible)
Top5 Euclidean ($Top5_{E}$)	93%	93%	66%
Top5 Cosine ($Top5_{C}$)	80%	80%	60%
Top3 Euclidean ($Top3_{E}$)	80%	73%	60%
Top3 Cosine ($Top3_{C}$)	80%	73%	60%

**Table 2:** Comparison of hyperspectral face recognition accuracy and compression ratio.

	Dataset			Extracted		Accuracy	Compression
	Dataset/Size	Bands	Spectrum	Features	Bands		Ratio
[28]	200	31	0.7–1.0 μm	5	31	75%	99.9995%
[29]	25 (PolyU)	33	0.4–0.72 μm	2700 (54 × 50)	24	78%	99.2509%
[27]	CMU	65	0.4–0.72 μm	69,169 (263 × 263)	65	86.1%	93.2264%
[24]	UWA	33	0.4–0.72 μm	900 (30 × 30)	4	98%	99.9895%
	PolyU	24	0.45–0.68 μm	1748 (46 × 38)	5	95.2%	99.8610%
[32]	PolyU	33	0.4–0.72 μm	4096 (64 × 64)	4	95%	99.8106%
	CMU	65	0.4–0.72 μm	4096 (64 × 64)	37	98%	99.7776%
Ours	UWA	33	0.4–0.72 μm	70	33	93%	99.9933%

**Table 3:** Comparison of recognition rates with state-of-art proposals.

Proposal	Method	All Face (100% ROI Visible)	Upper Part (50% ROI Visible)	Forehead (25% ROI Visible)
[18]	CNN+BoF	NA	91.3%	NA
[34]	CNN+SVM	NA	87%	NA
[33]	CNN	97.36%	95.38%	23.07%
[35]	PCA+SVM	90.14%	67.82%	NA
[36]	SRC	91.92%	72.37%	NA
[37]	CNN	97.21%	68.69%	NA
[37]	DGCAN+CNN	97.36%	75.21%	NA
Ours	AP+SVM (SVM)	93%	86.67%	40%

## Data Availability

M. Uzair, A. Mahmood and A. Mian. 2015. UWA Hyperspectral Face Database. https://openremotesensing.net/knowledgebase/uwa-hyperspectral-face-database-tip-2015-and-bmvc-2013/, accessed on 7 September 2022.
